# SOD1 Lysine 123 Acetylation in the Adult Central Nervous System

**DOI:** 10.3389/fncel.2016.00287

**Published:** 2016-12-20

**Authors:** Michael Kaliszewski, Austin K. Kennedy, Shelby L. Blaes, Robert S. Shaffer, Andrew B. Knott, Wenjun Song, Henry A. Hauser, Blaise Bossy, Ting-Ting Huang, Ella Bossy-Wetzel

**Affiliations:** ^1^Burnett School of Biomedical Sciences, College of Medicine, University of Central FloridaOrlando, FL, USA; ^2^Yale School of Forestry and Environmental Studies, Yale UniversityNew Haven, CT, USA; ^3^Department of Neurology and Neurological Sciences, Stanford University School of MedicineStanford, CA, USA; ^4^Geriatric Research, Education, and Clinical Center, VA Palo Alto Health Care SystemPalo Alto, CA, USA

**Keywords:** SOD1, acetylation, neurons, cerebellum, hippocampus, ventricle, olfactory bulb, retina

## Abstract

Superoxide dismutase 1 (SOD1) knockout (*Sod1*^−/−^) mice exhibit an accelerated aging phenotype. In humans, *SOD1* mutations are linked to familial amyotrophic lateral sclerosis (ALS), and post-translational modification (PTM) of wild-type SOD1 has been associated with sporadic ALS. Reversible acetylation regulates many enzymes and proteomic studies have identified SOD1 acetylation at lysine 123 (K123). The function and distribution of K123-acetylated SOD1 (Ac-K123 SOD1) in the nervous system is unknown. Here, we generated polyclonal rabbit antibodies against Ac-K123 SOD1. *Sod1* deletion in *Sod1^−/−^* mice, K123 mutation or preabsorption with Ac-K123 peptide all abolished antibody binding. Using immunohistochemistry, we assessed Ac-K123 SOD1 distribution in the normal adult mouse nervous system. In the cerebellum, Ac-K123 SOD1 staining was prominent in cell bodies of the granular cell layer (GCL) and Purkinje cell dendrites and interneurons of the molecular cell layer. In the hippocampus, Ac-K123 SOD1 staining was strong in the fimbria, subiculum, pyramidal cells and Schaffer collateral fibers of the cornus ammonis field 1 (CA1) region and granule and neuronal progenitor cells of the dentate gyrus. In addition, labeling was observed in the choroid plexus (CP) and the ependyma of the brain ventricles and central canal of the spinal cord. In the olfactory bulb, Ac-K123 SOD1 staining was prominent in axons of sensory neurons, in cell bodies of interneurons and neurites of the mitral and tufted cells. In the retina, labeling was strong in the retinal ganglion cell layer (RGCL) and axons of retinal ganglion cells (RGCs), the inner nuclear layer (INL) and cone photoreceptors of the outer nuclear layer (ONL). In summary, our findings describe Ac-K123 SOD1 distribution to distinct regions and cell types of the normal nervous system.

## Introduction

Superoxide dismutase 1 (SOD1) is an antioxidant enzyme that converts superoxide radicals to hydrogen peroxide and oxygen (McCord and Fridovich, 1969). SOD1 is a predominantly cytoplasmic enzyme that is ubiquitously expressed, conserved across species and abundant in most cell types of the nervous system (Crapo et al., 1992; Pardo et al., 1995). *Sod1* knockout (*Sod1)* mice exhibit various aging phenotypes that include retinal degeneration and vision loss, cochlear hair cell degeneration and hearing loss, accelerated motor neuron degeneration after axonal injury and exacerbated memory loss and amyloid deposition when crossed to a mouse model of Alzheimer’s disease (AD), raising the possibility that SOD1 may play an important neuroprotective role (Behndig et al., 2001; Imamura et al., 2006; Hashizume et al., 2008; Murakami et al., 2011, 2012; Kojima et al., 2012; Saccon et al., 2013).

Mutations in the human *SOD1* gene^1^ cause familial amyotrophic lateral sclerosis (ALS), a neurodegenerative disorder of motor neurons (Rosen et al., 1993). However, oxidation of SOD1 has also been linked to sporadic ALS (Rakhit et al., 2002), indicating that specific post-translational modifications (PTMs) of the wild-type protein may contribute to late-onset disease. Glutathionylation, palmitoylation and succinylation are among the characterized PTMs of SOD1 (Redler et al., 2011; Antinone et al., 2013; Lin et al., 2013). Several proteomic studies have also identified acetylation at conserved lysine 123 (K123; Choudhary et al., 2009; Zhao et al., 2010; Yang et al., 2011; Lundby et al., 2012; Weinert et al., 2013). K123 maps to the electrostatic loop of SOD1, a region required for copper binding, protein folding and substrate recruitment (Crapo et al., 1992; Pardo et al., 1995). Lysine acetylation is a reversible PTM that regulates the interaction, subcellular localization, folding and activity of many proteins (Kouzarides, 2000). The functional effects of lysine acetylation are wide ranging (Drazic et al., 2016). Dysregulation of lysine acetylation has been reported in numerous disorders including neurodegeneration and cancer (Drazic et al., 2016).

Here, we report the generation of new polyclonal rabbit antibodies against the K123-acetylated form of SOD1 and characterize the distribution of Ac-K123 SOD1 in the normal mouse nervous system.

## Materials and Methods

### Materials

Kanamycin A monosulfate, phosphate buffered saline (PBS), pH 7.4, EDTA, Tubastatin A hydrochloride, gelatin from cold water fish skin 45% in H_2_O, Ponceau S solution 0.1% w/v and 5% acetic acid, SOD assay kit, sodium orthovanadate, CuCl_2_, ZnSO_4_•H_2_O, poly-L-lysine, Mowiol 4–88 and 1,4-diazabicyclo-[2,2,2]-octane (DABCO) were obtained from Sigma-Aldrich. Trichostatin A (TSA) was obtained from Cayman Chemical. Nicotinamide (NAM) and MgSO_4_7•H_2_O were obtained was from Fluka Chemical. Dulbecco’s Modified Eagle Medium (DMEM) containing 4500 mg/L D-glucose, GlutaMAX-1, One Shot fetal bovine serum (FBS), Opti-MEM + GltaMAX/reduced serum medium, N-2 supplement, 100× penicillin-streptomycin solution, L15 and Earle’s Balanced Salt Solution (EBSS) were obtained from Gibco by Life Technologies. Bolt LDS sample buffer, Bolt 4%–12% Bis-Tris Plus polyacrylamide gels, Bolt MES sodium dodecyl sulfate (SDS) running buffer, Colloidal Blue staining kit and BL21(DE) competent cells were obtained from Invitrogen by Thermo Scientific. Premium cover glass was obtained from Fisher Scientific. Gene Jet Plasmid Maxiprep kit, Tissue Protein Extraction Reagent (T-PER), HALT^TM^ protease inhibitor cocktail and HALT^TM^ phosphatase inhibitor cocktail were obtained from Thermo Scientific. iBlot Dry Blotting System and iBlot Gel transfer stacks were obtained from Novex by Life Technologies. TurboFect transfection reagent was obtained from Fermentas. Neutralized bacteriological peptone was obtained from Oxoid. HEPES-free acid, isopropyl β-D-1-thiogalactopyranoside (IPTG), dithiothreitol (DTT), NaCl and KCl were obtained from OmniPur/Calbiochem. Sucrose-ultrapure was obtained from J.T. Baker. Tris-proteomic grade, β-mercaptoethanol-proteomic grade and Histochoice MB Tissue Fixative were obtained from AMRESCO. 3-[(3-Cholamidopropyl)dimethylammonio]-1-propanesulfonate (CHAPS) was obtained from G-Biosciences. Paraformaldehyde-EM grade, Prill purified was obtained from Ted Pella, Inc. Optimal Cutting Temperature (OCT) compound, Tissue-Tek Cryomolds and Accu-Edge low profile microtome blades were obtained from Sakura. Isoflurane, USP was obtained from Abbott Laboratories. Ten percent Tween-20 Surfact-Amps detergent solution, Pierce Coomassie Plus Bradford assay kit, Pierce enhanced chemiluminescence (ECL) Western Blotting Substrate, 20 mM Hoechst 33342 solution and Cytoseal 60 were obtained from Thermo Fisher Scientific. QuikChange Lightning site-directed mutagenesis kit was obtained from Agilent Technologies. PCR primers for site-directed mutagenesis were obtained from Integrated DNA Technologies. QIAprep Spin Miniprep Kit was obtained from Qiagen. Difco Luria Bertani broth (LB), Lennox, was obtained from Becton, Dickinson, and Company. Ethanol, agar, yeast extract and tryptone were obtained from EMD Millipore. Tissue culture dishes (100 and 145 mm) were obtained from Greiner Bio. Costar cell scrapers, Falcon polypropylene tubes, 75 cm^2^ Cellgro culture flasks and 0.25% trypsin were obtained from Corning Life Sciences. cOmplete Mini, EDTA-free protease inhibitors were obtained from Roche. Amersham Hyperfilm ECL high performance chemiluminescence film and DEAE Sephacel beads were obtained from GE Healthcare Limited. VECTASHIELD Antifade Mounting Medium was obtained from Vector Laboratories, Inc. Superfrost Plus micro slides were obtained from VWR. Twelve millimetre diameter #1 glass coverslips were obtained from Chemglass Life Sciences.

### Mice and rats

B6;129S-Sod1^tm1Leb^/J (*Sod1^−/+^*), B6126F1/J, (*Sod1^+/+^*), DBA/2J and 129S1/SvlmJ mice were obtained from Jackson Laboratories. B6.Cg-*Sod1^tm1Cje^* (*Sod1^−/−^*) mice were generated as previously described (Huang et al., 1997). Brain and eye tissue were collected from three 5-month-old male mice of both DBA/2J and 129S1/SvlmJ strains. Timed-pregnant Sprague Dawley rats were purchased from Charles River. All work with animals was performed in accordance with Institutional Animal Care and Use Committee (IACUC) of the University of Central Florida protocols.

### Antibodies

Custom polyclonal rabbit antibodies for Ac-K123 SOD1 were generated by YenZym Antibodies, LLC, by immunization of two rabbits (R25 and R26) with the peptide VVHE-acK-ADDLGKGGC (Ac-peptide) of human SOD1_119–131_ conjugated to the carrier protein THY. Pre-immune serum, test serum and final anti-serum were collected and antibody titers were confirmed by enzyme-linked immunosorbent assay (ELISA). Acetylation-specific antibodies were purified from anti-sera using an affinity matrix conjugated with the Ac-peptide. To eliminate any cross-reactive material, the antibody fraction was then further purified using a matrix conjugated with the unacetylated peptide VVHEKADDLGKGGC (peptide). Purification of the acetylation-specific antibodies was confirmed using ELISA with the acetylated and unacetylated peptide.

Commercial mouse monoclonal SOD1 (G-11), rabbit polyclonal SOD1 (FL-154), goat polyclonal calbindin D28K (N-18) and goat polyclonal doublecortin (C18) antibodies were obtained from Santa Cruz Biotechnology, Inc. Chicken polyclonal glial fibrillary acidic protein (GFAP; ab4674) antibody and rabbit polyclonal beta-III tubulin (ab18207) antibody were obtained from Abcam. Mouse monoclonal α-tubulin (DM1A) antibody was obtained from Cell Signaling Technology. Mouse monoclonal NeuN (A60) monoclonal antibody was obtained from EMD Millipore. Mouse monoclonal K40 acetylated-α-tubulin (6-11B-1) antibody was obtained from Sigma. Rabbit polyconal SOD1 (10011387) was obtained from Cayman Chemical.

Secondary peroxidase-conjugated AffiniPure donkey anti-rabbit IgG (H + L) peroxidase-conjugated AffiniPure donkey anti-mouse IgG (H + L), and Alexa Fluor 594 AffiniPure Fab fragment donkey anti-rabbit IgG (H + L) antibodies were obtained from Jackson ImmunoResearch Laboratories, Inc. Goat anti-rabbit IRDye 800CW and goat anti-mouse IRDye 680LT antibodies were obtained from LI-COR. Alexa Fluor 488 goat-anti-rabbit IgG (H + L), highly cross-absorbed, Alexa Fluor 594 goat anti-mouse IgG (H + L), Alexa Fluor 594-donkey anti-goat IgG (H + L) and Alexa Fluor 647 donkey-anti-chicken IgG (H + L) were obtained from Molecular Probes.

### Site-Directed Mutagenesis

Mutagenesis was performed using the QuikChange Lightning site-directed mutagenesis kit. The SOD1 K123Q and SOD1 K123R mutants for bacterial expression were created from a pET-3d SOD1 WT vector (gift from Dr. Joseph Beckman, Oregon State University). The EGFP-tagged SOD1-K123R mutant was created using the mammalian expression vector pEGFP-C1 WT SOD1 as a template (gift from Dr. Kurt J. DeVos, University of Sheffield). The primers utilized for mutagenesis were as follows:

**Table:** 

Primer	Sequence
K123Q Forward	5′-GCACACTGGTGGTCCATGAACAAGCAGATGACTTGGGC-3′
K123Q Reverse	5′-GCCCAAGTCATCTGCTTGTTCATGGACCACCAGTGTGC-3′
K123R Forward	5′-GCACACTGGTGGTCCATGAAAGAGCAGATGACTTGGGC-3′
K123R Reverse	5′-GCCCAAGTCATCTGCTCTTTCATGGACCACCAGTGTGC-3′

The DNA template-primer mix was amplified using an Eppendorf Mastercycler Gradient 531 PCR machine. After PCR amplification, template DNA was digested by incubation with DpnI at 37°C for 5 min. For bacterial transformation, 22.5 μL of XL10-Gold Ultracompetent bacteria were incubated with 2 μL of PCR-amplified DNA on ice for 30 min, heat-shocked at 42°C for 30 s, and placed back on ice for 2 min. Bacteria were then resuspended in 500 μL pre-warmed super optimal broth (SOB; 0.5% yeast extract, 2% tryptone, 10 mM NaCl, 2.5 mM KCl, 20 mM MgSO_4_) and then grown for 1 h at 37°C in a bacterial shaker at 225 rpm. Finally, 200 μL was plated on LB kanamycin A (100 μg/mL) agar plates. After 37°C overnight incubation, single colonies were picked and inoculated in 5 mL LB medium with kanamycin A (100 μg/mL). Bacterial cultures were grown overnight at 37°C in a bacterial shaker at 225 rpm. Plasmid DNA was isolated using a QIAprep Spin Miniprep kit and subjected to DNA sequencing to confirm mutagenesis (Eurofins Scientific). Large-scale endotoxin-free plasmid DNA was isolated using a Gene Jet Plasmid Maxiprep kit.

### Transfection and HEK293 Cell Extracts

HEK293 cells were grown at 5% CO_2_ and 37°C in 100 mm tissue culture dishes with 10 mL growth medium (DMEM containing 4500 mg/L D-glucose, GlutaMax-1, supplemented with penicillin/streptomycin and 10% FBS). Transfection was performed when cells reached ~80% confluency. pEGFP-C1-WT or pEGFP-C1 SOD1 mutant plasmid DNA (10 μg) was mixed with 20 μL TurboFect transfection reagent in 1 mL reduced serum Opti-MEM medium and incubated for 20 min at room temperature. The DNA/transfection mixture was then added drop-wise to the cells in growth medium. After 48 h, the cells were lysed and cell extracts were prepared. Briefly, the cells were placed on ice and the growth medium was aspirated and replaced with 5 mL ice-cold PBS. Cells were removed from the culture dish using a cell scraper and pelleted by centrifugation at 100× g for 10 min at 4°C in an Eppendorf 5810R table centrifuge. The cell pellets were then resuspended in 500 μL of ice-cold lysis buffer with 50 mM HEPES-Na pH 7.4, 150 mM NaCl, 1 mM EDTA, 2% CHAPS, 2 mM DTT, 1 mM MgCl_2_, 1 mM NAM, 1 μM TSA, 2 mM sodium orthovanadate and cOmplete EDTA-free protease inhibitors. After 30 min on ice and intermittent vortexing, soluble proteins were cleared by centrifugation at 18,200× g for 20 min at 4°C in an Eppendorf 5417R microcentrifuge. Supernatants were saved and protein concentrations were determined using a Pierce Bradford protein assay kit.

### Deacetylase Inhibition and HEK293 Cell Extracts

HEK293 cells seeded onto 145 mm culture dishes in 20 mL growth medium were grown to near confluency and treated with deacetylase inhibitors (10 mM NAM plus 1 μM TSA) or a DMSO vehicle control. After a 6-h treatment at 5% CO_2_ and 37°C, cells were harvested as described in 2.5 and resuspended in ice-cold Tissue Protein Extraction Reagent (T-PER) supplemented with 200 μM NAM, 10 μM TSA and HALT^TM^ protease and phosphatase inhibitors at a volume of 10 mL/g cell pellet. Cells were then subjected to three freeze/thaw cycles using a dry ice/ethanol bath and 37°C water bath followed by one cycle of sonication on ice (30 s at output 5 and 50% duty cycle) using a Branson Sonifer 450. Cellular debris was removed by centrifugation at 18,200× g for 20 min at 4°C in an Eppendorf 5417R microcentrifuge. Cleared supernatants were saved and protein concentrations were determined with a Pierce Bradford protein assay kit.

### Tissue Lysates

Isolated brains from *Sod1^−/−^* or *Sod1^+/+^* mice were frozen and stored at −70°C. Prior to tissue lysate preparation, brains were equilibrated to −20°C for 30 min, minced into small pieces and 1 g tissue was resuspended in 10 mL ice-cold T-PER Tissue Protein Extraction Reagent with 200 μM NAM, 10 μM TSA, 10 μM Tubastatin A and HALT^TM^ protease and phosphatase inhibitors. Following a 30-min incubation on ice, tissues underwent five cycles of sonication on ice (20 s at output 5 and 50% duty cycle) using a Branson Sonifer 450. Tissue lysates were then passed through a syringe fitted with a 25 1/2 gauge needle until no longer viscous. Cellular debris was removed by centrifugation at 18,200× g for 30 min at 4°C in an Eppendorf 5417R microcentrifuge. Supernatants were collected and protein concentrations were determined using a Pierce Bradford assay kit.

### Western Blotting

Protein samples (30 μg) were mixed with Bolt LDS sample buffer and 5% β-mercaptoethanol, heated at 95°C for 10 min, and loaded onto a Bolt 4%–12% Bis-Tris Plus gradient gel. Electrophoresis was carried out using Bolt MES SDS running buffer and a constant voltage of 120 V. Following electrophoresis, gels were soaked in ice-cold 20% ethanol in ddH_2_O for 10 min. Proteins were then transferred to nitrocellulose membranes (0.2 μm pore size) using iBlot Gel transfer stacks and an iBlot Dry Blotting System, program P3 for 10 min. Transfer efficiency was verified by staining with Ponceau S 0.1% w/v, 5% acetic acid solution. Membranes were briefly rinsed with ddH_2_O to remove the Ponceau S staining. To block unspecific protein binding, membranes were incubated with blocking buffer comprising 5% peptone, 1% gelatin in 20 mM Tris-Cl, pH 7.8, 150 mM NaCl and 0.1% Tween-20 Surfact-Amps (TBST) for 3 h at room temperature under constant rocking. Membranes were then rinsed with TBST and incubated overnight at 4°C with primary antibodies diluted in 1% peptone, 1% gelatin in TBST at 1 μg/mL for rabbit polyclonal Ac-K123 SOD1, 1:5000 for mouse monoclonal SOD1 (G11), 1:200 for rabbit polyclonal SOD1 (FL-154) and 1:2500 for mouse monoclonal α-Tubulin (DMA1). In antibody peptide preabsorption controls, Ac-K123 SOD1 antibody was mixed for 2 h at room temperature in 1% peptone, 1% gelatin in TBST with either Ac-K123 peptide, K123 peptide or a mixture of the two peptides. Immediately following preabsorption, the antibody/peptide mix was used for western blotting. Following primary antibody incubation overnight, membranes were washed 5× with 100 mL TBST for 5 min under rigorous shaking. For ECL western blotting, membranes were then incubated with secondary peroxidase-conjugated AffiniPure donkey anti-rabbit IgG (H + L) or peroxidase-conjugated AffiniPure donkey anti-mouse IgG (H + L) diluted 1:10,000 in 1% peptone, 1% gelatin in TBST for 2 h at room temperature and then washed 5× with 100 mL TBST for 5 min. Finally, membranes were rinsed once in TBS and protein-antibody complexes were visualized using a Pierce ECL Western Blotting Substrate and exposure to Amersham Hyperfilm ECL high performance chemiluminescence film. For infrared fluorescence western blotting, blocking and primary antibody incubation were carried out as described above. Secondary goat anti-rabbit IRDye 800CW or goat anti-mouse IRDye 680LT antibodies were diluted 1:20,000 in 1% gelatin in TBST and incubated light-protected for 2 h at room temperature under constant rocking. Membranes were washed 4× with 100 mL TBST and 1× with TBS. Protein-antibody complexes were then detected by scanning the membranes using a LI-COR Odyssey 9120 infrared imaging system. ImageJ software was used for densitometric analysis and statistical analysis was performed using Prism 6 (GraphPad).

### Primary Spinal Cord Astrocytes

Primary astrocytes were isolated from spinal cords of one litter of E18 embryos from a timed-pregnant Sprague Dawley rat. Spinal cord tissues were isolated, minced and digested in 0.025% trypsin in EBSS at 37°C for 20 min. Tissues were transferred into a 50 mL tube containing 45 mL pre-warmed astrocyte medium (DMEM containing 4500 mg/L D-glucose, GlutaMax-1, N-2 supplement, 10% FBS and penicillin/streptomycin) and allowed to sink. After tissues settled, medium was removed and replaced with 10 mL fresh astrocyte medium. Tissues were then triturated 20 times using a P1000 micropipette and seeded onto three 75 cm^2^ Cellgro culture flasks and cultured at 37°C, 5% CO_2_. Medium was replaced the next day and astrocytes were grown until confluent. Upon reaching confluency, astrocytes were trypsinized with 0.25% trypsin and seeded at 2 × 10^5^ cells/cm^2^ onto 12 mm diameter #1 glass coverslips coated with poly-L-lysine (50 μg/mL in ddH_2_O). Prior to immunostaining, confluent astrocytes were treated for 5 h with either deacetylase inhibitors (10 mM NAM, 1 μM TSA) or DMSO vehicle control.

### Immunocytochemistry

Astrocytes grown on glass coverslips were fixed in freshly prepared 4% paraformaldehyde in PBS pH 7.4 for 10 min at room temperature followed by three 5-min PBS washes. Fixed cells were permeabilized with 0.10% Triton X-100 in PBS for 10 min. Cells were then washed with PBS three times for 5 min. Blocking was performed by incubating fixed cells with 1% fish gelatin in PBS for 30 min at room temperature. Incubation with primary antibodies was performed in 1% fish gelatin in PBS at 4°C overnight in a humidified chamber. Primary antibodies included rabbit polyclonal Ac-K123 SOD1 R26 (1 μg/mL) and rabbit polyconal SOD1 (10011387; 1:2000). In antibody preabsorption controls, Ac-K123 SOD1 antibody was mixed for 2 h at room temperature in 1% cold fish gelatin in PBS with either Ac-peptide, unacetylated peptide or a mixture of the two peptides. Immediately following preabsorption, the antibody/peptide mix was used for immunocytochemical staining. Following primary antibody incubation, coverslips underwent three 5-min PBS washes. Goat-anti-rabbit Alexa Fluor 488 conjugated secondary antibody diluted 1:250 in 1% fish gelatin in PBS was then applied to coverslips for 1 h at room temperature within a humidified chamber. Nuclei were stained with 1 μg/mL Hoechst 33342 in PBS for 10 min. Coverslips underwent three 5-min PBS washes and were mounted onto VECTASHIELD Antifade Mounting Medium and sealed onto glass slides with Cytoseal 60. Mean fluorescence intensity measurements were made with ImageJ software and statistical analysis was performed using Prism 6 (GraphPad).

### Tissue Embedding

Tissues were collected following the guidelines of the IACUC. To isolate retinas, 129S1/SvlmJ mice were sacrificed by a lethal dose of isoflurane. Eyes were removed and radial cuts were made in the cornea to remove the lens. The isolated eye cups were then fixed in 4% paraformaldehyde, 5% sucrose, in PBS, for 15 min at room temperature. Retinas were then isolated and fixed for an additional 45 min in 4% paraformaldehyde, 5% sucrose, in PBS. After fixation, the retinas were rinsed 3× for 10 min with 5% sucrose and then 10% sucrose in PBS. Finally, the retinas were kept in 20% sucrose in PBS overnight at 4°C. For tissue embedding, retinas were incubated for 30 min in 100% OCT, followed by 30 min in one part 20% sucrose in PBS to two parts OCT, and then 30 min in two parts 20% sucrose in PBS to one part OCT. Finally, the retinas were embedded in two parts 20% sucrose in PBS to one part OCT in Tissue-Tek Cryomolds (25 mm × 20 mm × 5 mm) using a dry ice/2-methyl butane bath after the boiling stopped. Brains and spinal cords from 129S1/SvlmJ or DBA2/J mice were processed in the same manner, except that final embedding was performed in 100% OCT. Unfixed brains of *Sod1^+/+^* and *Sod^−/−^* mice were stored at −70°C and directly embedded in 100% OCT as described above.

### Cryostat Sectioning

Before cryosectioning, frozen tissue blocks were equilibrated to the cutting temperature for at least 1 h in the chamber of a Leica CM 1850 cryostat. Sections of 8−12 μm were obtained using a low profile microtome blade at −20°C to −22°C. Tissue sections were collected on Superfrost Plus micro slides, air-dried using a desiccator for 1 h at room temperature and stored at −70°C under desiccant until used for immunohistochemistry.

### Immunohistochemistry

Tissue sections were removed from −70°C and air-dried for 30 min at room temperature. Sections were rehydrated in PBS for 10 min at room temperature. Sections from *Sod1^−/−^* and *Sod1^+/+^* mice were fixed in 4% paraformaldehyde in PBS for 10 min at room temperature, followed by three 10-min washes with PBS. Sections were then permeabilized for 15 min in 0.1% or 0.15% Triton X-100 in PBS, followed by three 5-min washes in PBS, and incubated in blocking buffer 2% gelatin, 2% FBS in PBS for 60 min at room temperature. Primary antibodies were diluted in 1% gelatin in PBS at 1 μg/mL for rabbit polyclonal Ac-K123 SOD1, 1:50 for goat polyclonal calbindin D28K, 1:200 for goat polyclonal doublecortin C18, 1:250 for chicken polyclonal GFAP, 1:1000 for mouse monoclonal NeuN A60 and 1:20,000 for mouse monoclonal Ac-K40 α-tubulin (6-11B-1) and incubated overnight at 4°C in a humidified chamber. Negative controls included primary Ac-K123 SOD1 antibody omission, pre-immune serum incubation or antibody preabsorption with the Ac-K123 peptide used for immunization. Excessive primary antibody binding was removed by 5-min washes in PBS at room temperature. Secondary antibody Alexa Fluor 488-conjugated goat anti-rabbit IgG (H + L), Alexa Fluor 594-conjugated goat anti-mouse IgG (H + L), Alexa Fluor 594-conjugated donkey anti-goat IgG (H + L), Alexa Fluor 647-conjugated goat anti-chicken IgG (H + L) or Alexa Fluor 594-conjugated Fab Fragment donkey anti-rabbit IgG were diluted 1:200 in 1% gelatin in PBS and incubated with the sections for 2 h at room temperature in a humidified chamber protected from light. Excessive secondary antibody binding was removed by two 5-min washes with PBS. Nuclei were stained by incubation with 1 μg/mL Hoechst 33342 in PBS for 10 min followed by two 5-min washes with PBS. Finally, the sections were mounted with either VECTASHIELD Antifade Mounting Medium or Mowiol medium containing 2.5% DABCO using glass coverslips. Glass slides were sealed with Cytoseal 60 and stored at 4°C protected from light until proceeding to microscopy.

### Microscopy and Image Analysis

Imaging was carried out with a Zeiss LSM 710 Inverted Axio Observer confocal laser scanning microscope with six laser excitation lines (405, 458, 488, 514, 543 and 633 nm), a 34 channel QUASAR detector for spectral analysis, and several objectives including a EC Plan-Neofluar 10 × 0.3 numeric aperture (NA) air objective, Plan-Apochromat 20 × 0.8 NA air objective, EC Plan-Neofluar 40 × 1.3 NA oil objective and a Plan-Apochromat 63 × 1.4 NA oil objective. Image acquisition and processing were performed using Zen 2012 software from Zeiss. To visualize Hoechst 33342, the excitation/emission wavelength was 405/495 nm using the diode laser. For Alexa Fluor 488, the excitation/emission wavelength was 488/540 nm using the multiline argon laser. For Alexa Fluor 594, the excitation/emission wavelength was 543/660 nm using a diode pumped solid state laser plus a QUASAR detector for all emission wavelengths. On average 25 *z*-stacks of 0.5 μm step-size were acquired for 354 μm × 354 μm scaled image size. The *z-stacks* were maximally projected and the gamma value adjusted to 0.70−0.85 using the ZEN 2012 software.

Montage images were acquired using a Zeiss Axiovert Zeiss 100M fluorescence microscope with a Plan-Neofluar 10 × 0.3 NA or Plan-Neofluar 20 × 0.5 NA air objective (Zeiss), a DG-4/ Xe-arc illumination unit (Sutter Instruments), a BioPrecision 2 linear encoded XY-motorized stage operated by a MAC 5000 modular control system (Ludl), and a Sensicam QE CCD camera (PCO AG) controlled by MetaMorph 7.1 software (Molecular Devices). To visualize Ac-K123 SOD1- Alexa 488, the excitation filter was S490/20× (Chroma) and the emission filter was S528/38 m (Chroma). To generate the montage, the “scan slide” function in Metamorph 7.1 was used. At each z position in the scan slide area, a three dimensional image was acquired using the “stream Z” module, generating an average of 100−400 image tiles (1 × 1 binning, 4 μm step size, 11 *z*-planes). The *z-stacks* were processed in MetaMorph 7.5 using the “remove haze” function and maximally projected. Finally, the montage was stitched with a 10% image overlay using the “make montage” function.

### Bacterial Expression of Human SOD1 Protein

BL21 (DE3) *E. coli* competent cells were transformed with pET-3d SOD1 WT, pET-3d SOD1 K123R or pET-3d SOD1 K123Q DNA by heat shock. Briefly, DNA (50 ng) was added to 100 μL of competent cells in pre-chilled Falcon polypropylene tubes and incubated on ice for 30 min. Heat shock was then performed in a 42°C water bath for 45 s followed by a 2-min incubation on ice. Transformed bacteria were then resuspended in 1 mL SOC medium and grown at 37°C for 1 h while shaking at 225 rpm. One hundred micro litre of bacterial suspension was then plated onto LB agar plates containing 100 μg/mL ampicillin and grown overnight at 37°C. Colonies were rinsed off the agar plates using 5 mL LB medium and used to inoculate a 1 L flask containing 250 mL LB media with 100 μg/mL ampicillin. Bacteria were then grown at 37°C while shaking at 225 rpm until OD_600_ values reached between 0.4 and 0.6. Protein expression was induced with 1 mM IPTG and grown for 3 h at 23°C with shaking at 150 rpm. At the time of induction of protein expression, cultures were supplemented with 0.05 mM CuCl_2_ and 0.1 mM ZnSO_4_•H_2_O.

### Purification of Human SOD1 from Bacteria

Transformed BL21 (DE3) *E. coli* expressing WT, K123R or K123Q mutant of human SOD1 as described in 2.15 above were collected by centrifugation at 4000× g for 10 min. Cell pellets were resuspended in 20 mM Tris-HCl pH 8 at 5 mL per gram and lysed by three quick freeze/thaw cycles using a dry ice/ethanol bath and 37°C water bath followed by three cycles of sonication (30 s at output 5 and 50% duty cycle) on ice. Lysates were cleared by centrifugation at 18,000× g for 20 min at 4°C and supernatants were then transferred to DEAE Sephacel columns where they were allowed to bind overnight at 4°C. SOD1 was eluted by NaCl gradient, starting at 0 mM NaCl and ending at 200 mM NaCl in 20 mM Tris-HCl pH 8.0. Collected fractions were evaluated by SDS-PAGE using a Colloidal Blue staining kit, and SOD1-containing fractions were combined and quantified by Bradford assay.

### Superoxide Dismutase Activity

To measure dismutase activity of human SOD1 WT, K123R and K123Q mutant protein expressed in bacteria, a SOD assay kit was used following manufacturer’s instructions.

## Results and Discussion

### SOD1 Deletion Abolishes Antibody Binding

**Figure 1:**
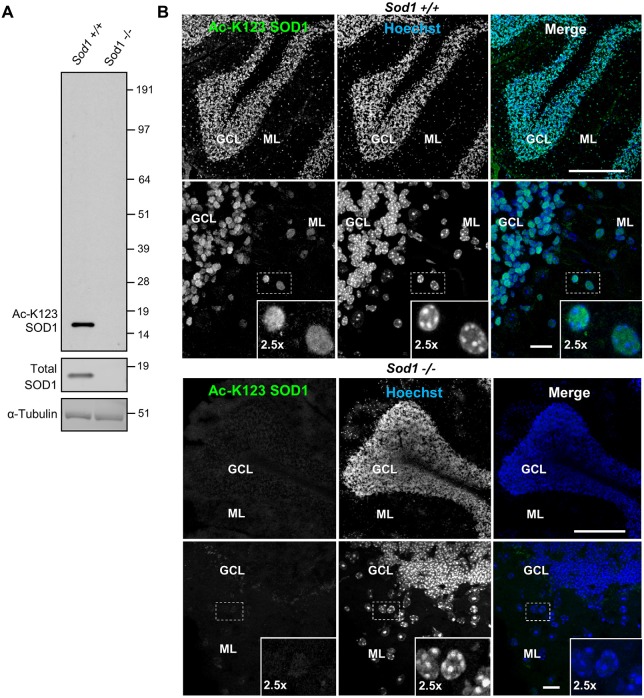
**Superoxide dismutase 1 (SOD1) knockout abolishes lysine 123-acetylated SOD1 (Ac-K123) antibody binding. (A)** Western blot of brain tissue lysates from *Sod1^+/+^* and *Sod1^−/−^* mice probed with Ac-K123 SOD1 (R25), SOD1 (FL-154) and α-Tubulin (DMA1) antibodies. **(B)** Confocal micrographs (scale bar, 200 μm) of *Sod1^+/+^* and *Sod1^−/−^* mouse cerebellum sagittal sections showing Ac-K123 SOD1 (R26) immunostaining, nuclear Hoechst 33342 labeling or merged image. The granular cell layer (GCL) and molecular layer (ML) of the cerebellar cortex are identified. Below each image is a higher magnification confocal micrograph (scale bar, 10 μm) of the GCL and ML. Insets show a 2.5× zoom of the boxed region.

To investigate the role of K123 acetylation of SOD1, we generated polyclonal rabbit antibodies. Two rabbits (R25 and R26) were immunized with the Ac-peptide representing SOD1_119–131._ Acetylation-specific antibodies were purified from anti-sera using an affinity matrix conjugated with the Ac-peptide used for immunization. To test whether the antibodies were specific for SOD1 and lacked cross-reactivity with unrelated proteins, brain tissue lysates of *Sod1^+/+^* and *Sod1*^−/−^ mice were separated by SDS-PAGE and western blot membranes were probed with primary antibodies for Ac-K123 SOD1, total SOD1 or α-tubulin. Protein bands were identified using the ECL detection system. The Ac-K123 SOD1 antibodies detected a single protein band corresponding to SOD1 in* Sod1*^+/+^ mice (Figure 1A, left lane). By contrast, *Sod1* gene deletion in *Sod1*^−/−^ mice abolished Ac-K123 SOD1 antibody binding (Figure 1A, right lane). Probing with total SOD1 antibodies confirmed *Sod1* gene deletion in *Sod1^−/−^* mice and α-tubulin antibodies demonstrate equal protein loading. The results indicate that our new antibody is specific to SOD1 and lacks cross-reactivity with unrelated proteins using western blotting techniques.

Next, we tested whether our antibody also lacked cross-reactivity using immunohistochemistry. Brain tissue sections from *Sod1*^−/−^ or *Sod1*^+/+^ mice were stained with primary Ac-K123 SOD1 antibodies and secondary Alexa-Fluor 488 conjugated antibodies. Nuclei were stained with Hoechst 33342 dye. Figure 1B shows confocal micrographs of the cerebellum. In *Sod1*^+/+^ mice, our custom antibody stained cell bodies of the granular cell layer (GCL) and molecular layer (ML; Figure 1B, top). By contrast, in *Sod1*^−/−^ mice no immunostaining was detected (Figure 1B, bottom). These results suggest that our custom antibodies are specific to SOD1 and can be used for immunohistochemistry.

### Antibodies Are Specific for K123-Acetylated SOD1

**Figure 2:**
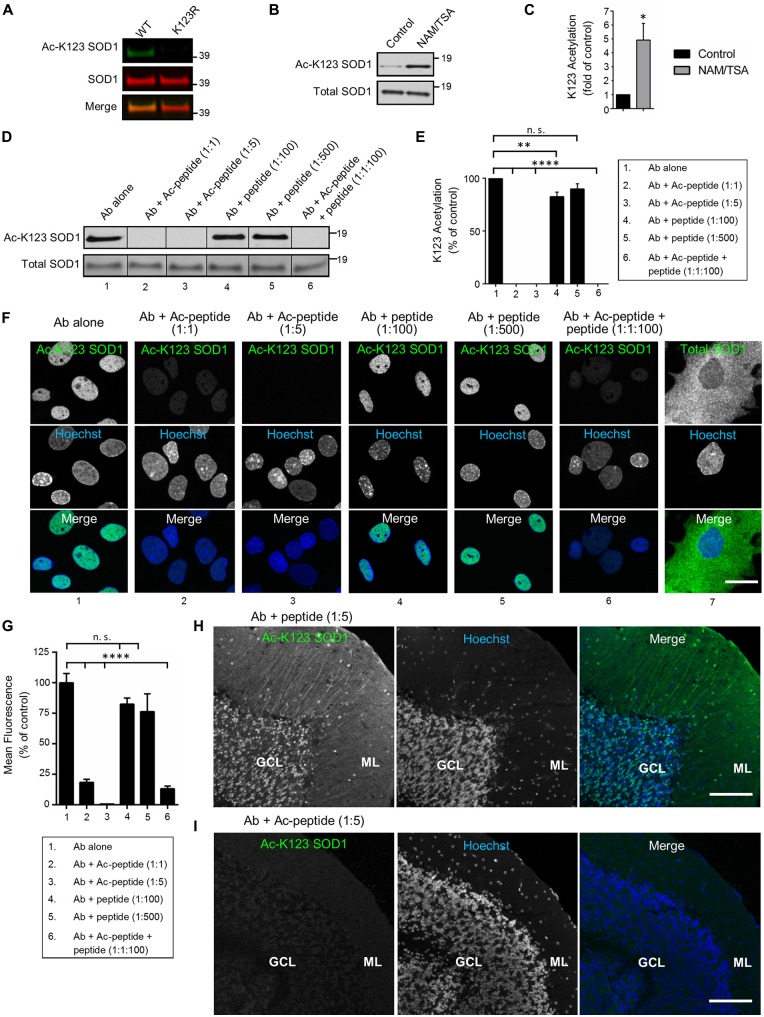
**Antibodies are specific for K123-acetylated SOD1. (A)** Infrared fluorescence western blot analysis of transfected HEK293 cells expressing SOD1 WT-EGFP or SOD1 K123R-EGFP. Ac-K123 SOD1 (R26) rabbit polyclonal antibody was detected by goat anti-rabbit IRDye 800CW (shown in green) and SOD1 (G-11) mouse monoclonal antibody was detected by goat anti-mouse IRDye 680LT antibodies (shown in red). **(B)** Enhanced chemiluminescence (ECL) western blot of lysates from HEK293 cells (pretreated for 6 h with DMSO vehicle control or nicotinamide (NAM)/trichostatin A (TSA)) probed with Ac-K123 SOD1 rabbit polyclonal or SOD1 (G-11) mouse monoclonal antibodies. Protein bands were detected by ECL. **(C)** Bar graph of quantification for Ac-K123 SOD1 densitometry normalized to SOD1. The acetylation level of NAM/TSA treated cells are plotted relative to vehicle control cells (*n* = 3, *significance at *P* < 0.05 by Student’s *t*-test).** (D)** Western blot analysis of protein extracts isolated from NAM/TSA treated HEK293 cells probed with Ac-K123 SOD1 (R26) antibody that was pre-incubated with either no peptide, Ac-peptide (1:1 or 1:5 molar ratio), unmodified peptide (1:100 or 1:500) or a mixture of Ac-peptide and unacetylated peptide (1:1:100). **(E)** Quantification of the antibody preabsorption western blot results. Ac-K123 SOD1 band intensities were normalized to those of SOD1 and plotted as percentage of K123 acetylation relative to the antibody alone (*n* = 3, **and significance at *P* < 0.01 and *P* < 0.0001 by ANOVA, respectively). **(F)** Confocal micrographs (scale bar, 20 μm) of primary spinal cord astrocytes immunostained with Ac-K123 SOD1 (R26) antibody that was pre-incubated with either no peptide, Ac-peptide, unacetylated peptide or a mixture of Ac-peptide and unacetylated peptide (the same molar rations as in 2D); or a commercial antibody for total SOD1. Hoechst 33342 nuclear labeling and merged images are also shown.** (G)** Quantification of antibody preabsorption immunocytochemistry results. Data are reported as percentage of nuclear mean fluorescence intensity relative to the antibody alone control of 150 (antibody alone), 100 (Ab + Ac-peptide 1:1), 100 (Ab + Ac-peptide 1:5), 100 (Ab + unacetylated peptide 1:100), 100 (Ab + unacetylated peptide 1:500) and 100 (Ab + Ac-peptide 1:1 + unacetylated peptide 1:100) astrocytes in three independent experiments (****significance at *P* < 0.0001 by ANOVA). **(H)** Confocal micrograph (scale bar, 100 μm) of cerebellum immunostained with Ac-K123 SOD1 (R26) antibody pre-incubated with unacetylated peptide at 1:5 molar ratio prior to immunohistochemistry. GCL, granular cell layer; ML, molecular layer. **(I)** Confocal micrograph (scale bar, 100 μm) of cerebellum immunostained with Ac-K123 SOD1 (R26) antibody preabsorbed with Ac-peptide at 1:5 molar ratio prior to immunohistochemistry. GCL, granular cell layer; ML, molecular layer.

To examine antibody specificity to the K123 acetylated form of SOD1, we transfected HEK293 cells with expression vectors encoding SOD1 WT or the K123R mutant (where lysine was replaced by an arginine making it resistant to acetylation). To differentiate from the endogenous protein, SOD1 was tagged with EGFP. After 48 h of expression, protein lysates were generated, separated by SDS-PAGE, and processed by infrared fluorescence western blotting. Transiently expressed SOD1 bands were easily distinguishable because endogenous SOD1 has a molecular weight of approximately 16 kDa while EGFP-tagged SOD1 has a molecular weight of 43 kDa. Infrared images indicated EGFP-SOD1 WT was recognized by both Ac-K123 SOD1 and SOD1 antibodies, as demonstrated by the overlapping bands in the merged image (Figure 2A, left lane). By contrast, the acetyl-resistant EGFP-SOD1 K123R mutant was recognized by SOD1 antibodies but not Ac-K123 SOD1 antibodies (Figure 2A, right lane). Thus, the inability to recognize SOD1 K123R suggests that the antibody is specific to the K123 acetylated form of SOD1.

Next, because SOD1 is also succinylated at K123 (Lin et al., 2013), we needed to confirm that our antibody recognized K123 acetylation and not another PTM. To do this, we assessed the detection of endogenous SOD1 K123 acetylation in HEK293 cells treated with either vehicle control or a mix of two pan-deacetylase inhibitors (NAM/TSA). After 6 h, cells were lysed and proteins were resolved by SDS-PAGE for western blot analysis. Vehicle-control treated cells exhibited a weak signal for Ac-K123 SOD1 (Figure 2B, left lane). By contrast, deacetylase inhibition dramatically increased the Ac-K123 SOD1 signal (Figure 2B, right lane). Reprobing the blot with antibodies for total SOD1 indicated that the amount of SOD1 was similar in both samples (Figure 2B). To quantify the results, we performed densitometric analysis of three independent western blots and observed a four-fold increase in Ac-K123 SOD1 in deacetylase inhibited cells (Figure 2C). Taken together, these data support that the increased signal detected in deacetylase inhibitor treated cells is due to increased Ac-K123 SOD1 levels, and that the antibody recognizes acetylation of K123.

Similar to other proteins modified by lysine acetylation, only a small fraction of total SOD1 is predicted to be acetylated (Choudhary et al., 2014). Therefore, despite affinity purification (see “Antibodies” Section), it was important to verify the absence of antibody cross-reactivity with unacetylated SOD1. To assess any cross-reactivity, we performed western blots of deacetylase inhibitor treated lysate using antibody that was pre-incubated with no peptide, acetylated peptide (Ac-peptide), unacetylated peptide (peptide) or a mixture of Ac-peptide and peptide. Pre-incubation of the antibody alone resulted in a strong signal for Ac-K123 SOD1 (Figure 2D, lane 1). As expected, preabsorption at 1:1 or 1:5 (antibody:Ac-peptide) molar ratios totally eliminated antibody binding to Ac-K123 SOD1 (Figure 2D, lanes 2, 3). By contrast, pre-incubation with peptide at 1:100 or 1:500 failed to eliminate antibody binding (Figure 2D, lanes 4, 5). Finally, pre-incubation with a mixture of antibody to Ac-peptide to peptide (1:1:100) also resulted in loss of signal by western blot (Figure 2D, lane 6). Quantification of three independent western blots using densitometry confirmed the results (Figure 2E). Combined, these western blot results confirm antibody specificity to Ac-K123 SOD1 and demonstrate an absence of cross-reactivity to unacetylated SOD1.

Next, it was important to show that the antibody does not cross-react with unacetylated SOD1 using other techniques such as immunocytochemistry. Therefore, we performed similar antibody-peptide pre-incubation experiments using rat primary spinal cord astrocytes. In the antibody alone condition, we observed immunostaining of Ac-K123 SOD1 antibody in the nucleus of astrocytes (Figure 2F, column 1). Antibody incubated with Ac-peptide at 1:1 or 1:5 molar ratio blocked immunoreactivity (Figure 2F, columns 2, 3). Preabsorption of antibody with unacetylated peptide at 1:100 or even 1:500 molar ratios failed to eliminate immunoreactivity (Figure 2F, columns 4, 5). Finally, antibody incubated with Ac-peptide and unacetylated peptide at 1:1:100 molar ratios failed to show immunoreactivity (Figure 2F, column 6). Immunostaining for total SOD1 using a commercial antibody revealed cytoplasmic SOD1 staining (Figure 2F, column 7). Quantification of mean fluorescence intensity is shown in Figure 2G.

Finally, to confirm antibody specificity using immunohistochemical techniques, we probed cerebellar tissue sections with Ac-K123 SOD1 antibodies pre-incubated with the unacetylated peptide or Ac-peptide at 1:5 molar ratios. Tissue sections probed antibodies pre-incubated with unacetylated peptide resulted in labeling of cell bodies of the GCL and cell bodies and processes of the ML (Figure 2H). By contrast, immunostaining was abolished in tissue sections probed with antibodies preabsorbed with Ac-peptide (Figure 2I). Similarly, primary antibody omission or incubation with pre-immune serum resulted in a lack of staining (Supplementary Figure 1).

Our finding that Ac-K123 SOD1 was localized to the nucleus was unexpected, as SOD1 is regarded as a predominantly cytoplasmic protein. SOD1 translocation to the nucleus has been previously described (Tsang et al., 2014), but our finding that nuclear SOD1 is acetylated at K123 is novel. Acetylation of K123 may play a role in SOD1 targeting or function in the nucleus. Of note, the K123 acetylation status did not impact enzyme activity as purified WT or mutant SOD1 (K123R, K123Q) all exhibited comparable SOD activities (Supplementary Figure 2).

In summary, antibody blocking with Ac-peptide prevented signal detection by western blot, immunocytochemistry of fixed cells and immunohistochemistry of brain tissue sections. These findings demonstrate that our custom antibodies are specific to K123 acetylated SOD1 and lack cross-reactivity to unacetylated SOD1. For the above reasons, we believe the results obtained below using this antibody likely reflect authentic Ac-K123 SOD1 in tissue.

### Ac-K123 SOD1 Is Found in Granular Cell Somata and Neurites of the Cerebellum

**Figure 3:**
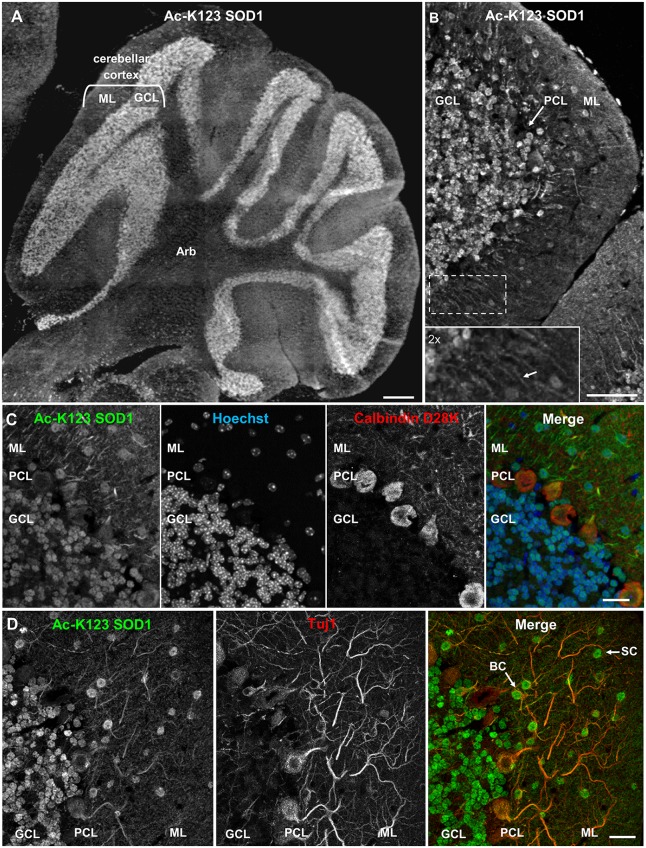
**Ac-K123 SOD1 localization within granule, basket and stellate cells (SC) somata and ML neurites of the cerebellum. (A)** Sagittal view micrograph montage (scale bar, 500 μm) of cerebellum showing Ac-K123 SOD1 (R26) immunostaining within the cerebellar cortex and underlying arbor vitae (Arb). The cerebellar cortex GCL and ML are noted.** (B)** Confocal micrograph (scale bar, 50 μm) demonstrating Ac-K123 SOD1 (R26) immunostaining in the cerebellum. Shown is the cerebellar cortex layers, consisting of the GCL, Purkinje cell layer (PCL) and ML. Inset shows a 2× zoom of the boxed region demonstrating Ac-K123 SOD1 labeled processes within the ML (arrow). **(C)** Higher magnification confocal micrograph (scale bar, 20 μm) of GCL, PCL and ML immunostained for Ac-K123 SOD1 (R26) and calbindin D28K with nuclear counterstaining by Hoechst 33342. **(D)** Confocal micrograph (scale bar, 20 μm) of GCL, PCL, and ML immunostained for Ac-K123 SOD1 (R26) and Tuj1. Examples of SC and basket cells (BC) are marked by arrows.

To investigate the distribution of Ac-K123 SOD1 in the cerebellum, we immunostained sagittal brain sections. Strong Ac-K123 SOD1 labeling was detected in the GCL (Figure 3A). The ML exhibited less intense labeling. A higher magnification view allowed evaluation of Ac-K123 SOD1 distribution in the GCL, Purkinje cell layer (PCL), and ML. The most intense immunostaining was observed in the cell bodies of small, densely packed glutamatergic neurons of the GCL (Figure 3B). Weak staining was observed in cell bodies of the PCL. Finally, staining was also observed in cell bodies and processes extending through the ML (Figure 3B, inset).

To further examine Purkinje cell staining, tissue was co-stained with antibodies against calbindin D28K, a Purkinje cell marker. Previous immunohistological examination demonstrated that SOD1 was present throughout all cortical layers of the cerebellum, including cell bodies, dendrites and axons of Purkinje cells (Thaete et al., 1986; Moreno et al., 1997; Lindenau et al., 2000). Interestingly, we observed weak Ac-K123 SOD1 labeling within the somata of Purkinje cells compared to cells of other cortical layers (Figure 3C).

We next focused on the distribution of Ac-K123 SOD1 within the ML. This outermost layer of the cerebellar cortex comprises granule cell axons, Purkinje cell dendritic arbors, basket and stellate cells (SC) interneurons and glial cells. To identify neurons, sections were co-stained with antibodies against the neuron-specific class III β-tubulin (Tuj1). We found Ac-K123 SOD1 localized to the cell bodies of ML interneurons (Figure 3D). The cerebellar SC, which are located in outer ML regions and synapse onto the dendritic arbors of Purkinje cells and basket cells (BC), located immediately above the PCL and forming elaborate branches around Purkinje cell bodies, were identified by both location and positive labeling for Tuj1 (Figure 3D). Labeling was also observed within neuronal processes extending into the ML, demonstrating Ac-K123 SOD1 distribution to Purkinje cell dendritic arbors and possibly the axons of granule cells (Figure 3D).

In summary, cerebellar immunostaining demonstrated strong Ac-K123 SOD1 labeling in the somata of granule cells and ML interneurons. Purkinje cell bodies exhibited less pronounced Ac-K123 SOD1 staining, as did neuronal processes extending into the ML.

### Ac-K123 SOD1 Localizes to Hippocampal Pyramidal Neurons and Granule Cells of the Dentate Gyrus

**Figure 4:**
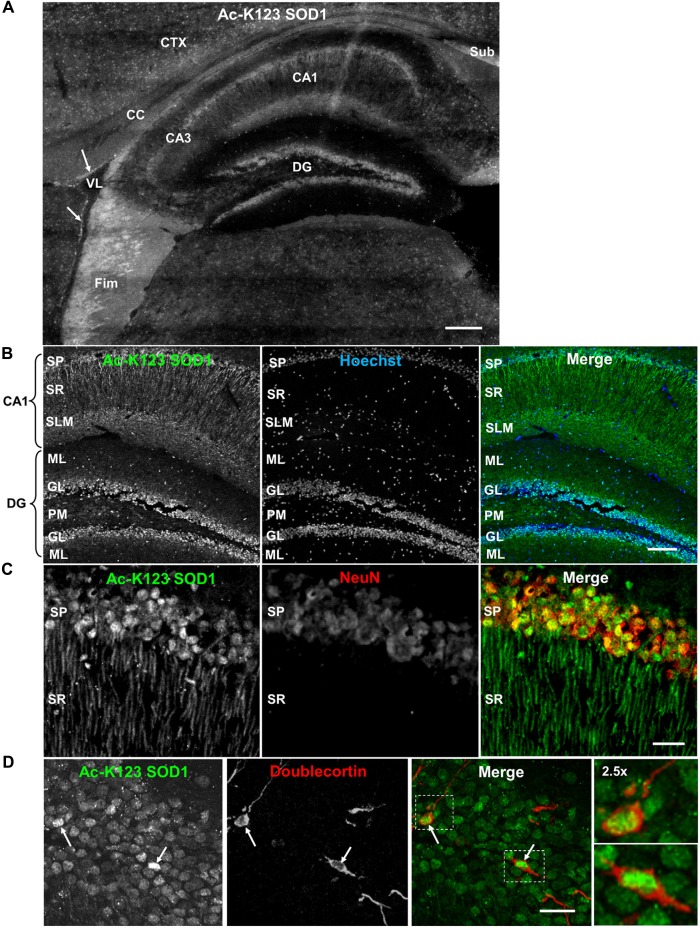
**Distribution of Ac-K123 SOD1 within dentate gyrus granule cells and cornus ammonis field 1 (CA1) hippocampal neurons. (A)** Sagittal view micrograph montage (scale bar, 200 μm) of the hippocampal region immunostained with Ac-K123 SOD1 (R26) antibodies. Areas of interest include the subiculum (Sub), fimbria (Fim), corpus callosum (CC), cortex (CTX), lateral ventricle (VL), dentate gyrus (DG), hippocampus CA1 and hippocampus CA3 field (CA3). Ac-K123 SOD1 labeling within cells lining the ventricle wall is also shown (arrows). **(B)** Confocal micrograph (scale bar, 100 μm) of the dentate gyrus and CA1 field of the hippocampus proper labeled with Ac-K123 SOD1 (R26) antibodies and Hoechst 33342. Dentate gyrus regions include the granule layer (GL), polymorphic layer (PM) and ML. The CA1 layers shown are the stratum pyramidale (SP), stratum radiatum (SR) and stratum lacunosum-moleculare (SLM). **(C)** Confocal micrograph (scale bar, 20 μm) of hippocampus CA1 field pyramidal neurons immunostained with antibodies against Ac-K123 (R26) SOD1 and the adult neuron specific marker NeuN. **(D)** Confocal micrograph (scale bar, 20 μm) showing the GL of the dentate gyrus immunostained with antibodies against Ac-K123 SOD1 (R26) and doublecortin. Immature adult granule neurons are noted (arrows). Insets show a 2.5× zoom of the boxed regions of merged image.

Next, we analyzed Ac-K123 distribution within the hippocampus and dentate gyrus by immunostaining sagittal brain sections with Ac-K123 SOD1 antibodies. A micrograph montage of the hippocampal region showed Ac-K123 SOD1 immunostaining was most prominent in the fimbria and subiculum (Figure 4A). Within the hippocampus labeling of cornus ammonis field 1 (CA1) was observed, while cornus ammonis field 3 (CA3) stained less strongly (Figure 4A). Staining was also observed in the dentate gyrus, the major target of cortical input to the hippocampus. Cells lining the lateral ventricle and the cortex were also labeled, and diffuse staining was observed in the corpus callosum (CC; Figure 4A).

Higher magnification of the hippocampus indicated Ac-K123 SOD1 localization to CA1 stratum pyramidale (SP) cell bodies and processes within the CA1 stratum radiatum (SR; Figure 4B). The CA1 SP mostly consists of pyramidal neurons, which receive input from Schaffer collaterals and send axons to the subiculum and entorhinal cortex. Also present within this layer, at a much lower density, are the somata and processes of local circuit inhibitory interneurons that include axo-axonic, basket, bistratified and radial trilaminar cells. The CA1 stratum lacunosum-moleculare (SLM), where performant pathway fibers from the entorhinal cortex synapse onto apical dendrites of CA1 pyramidal cells, also exhibited strong labeling (Figure 4B). Staining was also observed in the dentate gyrus within granule cells and in cell bodies of the ML and polymorphic layer (Figure 4B).

We examined the CA1 at higher magnification to assess labeling of pyramidal neuron cell bodies and processes of the SR. Co-labeling with antibodies against NeuN, a neuron-specific marker, confirmed Ac-K123 SOD1 localization to neuronal cell bodies within the SP (Figure 4C). These labeled cell bodies are tightly packed and layered in a characteristic pattern for pyramidal neurons of the hippocampus. A characteristic of CA1 pyramidal cells is the presence of a single apical dendrite that branches into secondary oblique dendrites farther away from the soma. Immunostaining results demonstrated Ac-K123 SOD1 distribution to the apical dendrites extending from pyramidal neurons into the CA1 SR (Figure 4C). Interestingly, labeling was not observed in any dendritic branches emanating from the apical dendrites of pyramidal neurons. Based on the closely arranged somata in the stratum pryamidale and the homogenous morphology of processes that extend into the SR, the observed Ac-K123 labeling within these layers can mostly, if not exclusively, be attributed to pyramidal neurons.

Next, we assessed immunostaining of the dentate gyrus granule layer cell bodies at higher magnification. Interestingly, Ac-K123 SOD1 labeling did not appear to be uniform (Figure 4D). Because of this uneven Ac-K123 SOD1 staining pattern of granule cells and the important role of this region in adult neurogenesis (Ming and Song, 2011), we sought to explore any possible relationship between Ac-K123 SOD1 and neuronal development. To examine Ac-K123 SOD1 in cells undergoing neurogenesis, brain tissue was co-labeled with antibodies against doublecortin, a marker for adult neurogenesis. Doublecortin expression is transient and tapers off in mature neurons (Brown et al., 2003). Ac-K123 SOD1 immunostaining was strongest in cell bodies of doublecortin-positive cells of the dentate gyrus (Figure 4D).

In summary, immunostaining of the hippocampus resulted in strong Ac-K123 SOD1 labeling of neuronal tracts, including the subiculum and fimbria. The neurons within the hippocampus and dentate gyrus, the pyramidal and granule cells, also stained for Ac-K123 SOD1. CA1 pyramidal cells of the hippocampus exhibited Ac-K123 SOD1 localization to cell bodies and dendrites. SOD1 carries out important neuromodulatory functions within the hippocampus. Secreted SOD1 modulates synaptic transmission and inhibits long-term potentiation, the long-lasting form of synaptic enhancement linked to learning and memory, within the dentate gyrus and CA1 region 1 of the hippocampus (Knapp and Klann, 2002; Viggiano et al., 2012). SOD1 deficiency exacerbates memory loss and amyloid deposition in a mouse model of AD, and SOD1 levels are significantly decreased in human AD patients (Murakami et al., 2011). Thus, it will be interesting to investigate the potential role of SOD1 acetylation in these processes. Ac-K123 SOD1 was also found in the somata of dentate gyrus granule cell layer and was highest in doublecortin-positive progenitor cells.

### Ac-K123 SOD1 Is Present in Ependymal and Choroid Plexus Cells

**Figure 5:**
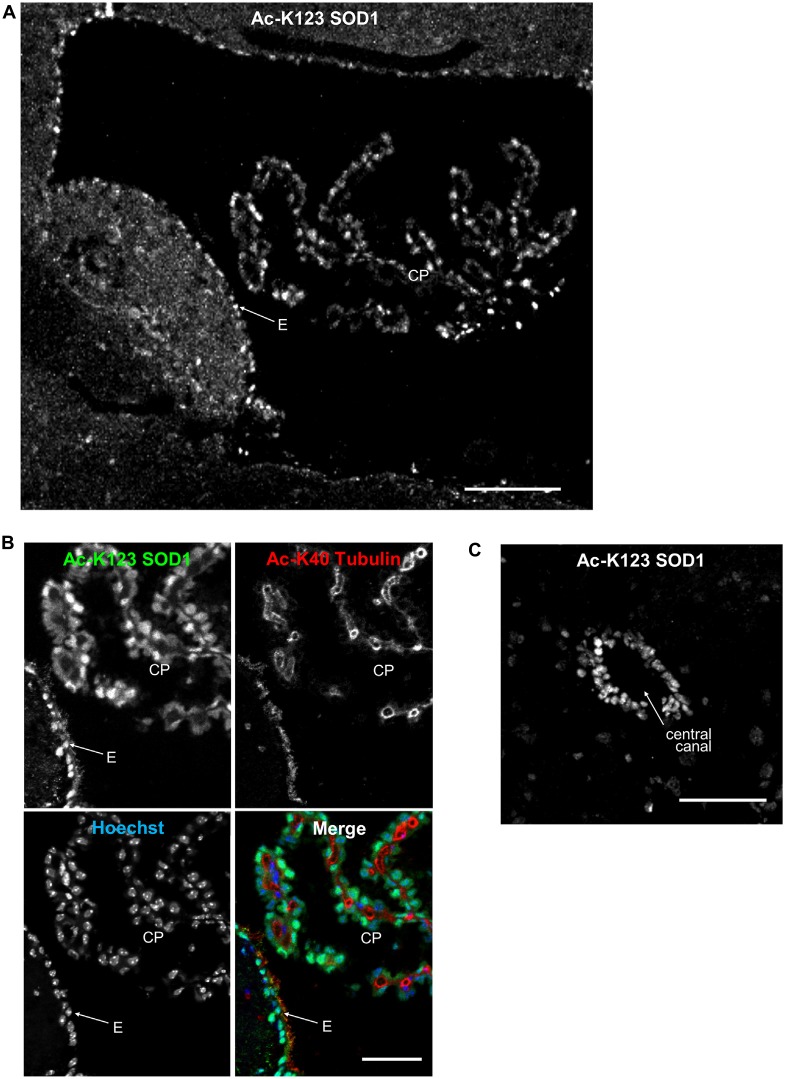
**Ac-K123 SOD1 localization in choroid plexus (CP) and ependymal cells. (A)** Sagittal view confocal micrograph (scale bar, 100 μm) of fourth ventricle immunostained for Ac-K123 SOD1 (R26). Identified are the CP and ventricle wall ependymal cells (E). **(B)** Higher magnification confocal micrograph (scale bar, 50 μm) of CP and E immunostained for Ac-K123 SOD1 (R26) and Ac-K40 α-Tubulin. Nuclei were counterstained with Hoechst 33342. **(C)** Confocal micrograph (scale bar, 50 μm) of a transverse section of spinal cord immunostained for Ac-K123 SOD1 (R26).

Recent findings have identified SOD1 in human cerebral spinal fluid (CSF; Winer et al., 2013). Thus, we sought to explore whether Ac-K123 SOD1 is present in specific regions of the brain in contact with CSF. CSF is produced by the four choroid plexus (CP), branched epithelial structures found within each ventricle. Analysis of immunostained sagittal sections demonstrated Ac-K123 SOD1 labeling of CP cells within the 4th ventricle (Figure 5A). Ac-K123 SOD1 labeling was also observed in ependymal cells lining the ventricle wall (Figure 5A). A higher magnification view confirmed strong SOD1 K123 acetylation within epithelial cells of the CP and ependymal cells (Figure 5B). Finally, staining was also observed in the ependymal cells lining the central canal of the spinal cord (Figure 5C).

These findings demonstrating Ac-K123 SOD1 distribution within ependymal cells and the CP epithelia merit further investigation into the role of SOD1 K123 acetylation in the regulation of CSF.

### Ac-K123 SOD1 Is Present in Interneuron Cell Bodies and the Major Afferent and Efferent Neuronal Processes of the Olfactory Bulb

Olfactory bulb alterations have been observed in mutant SOD1 transgenic mice (Dal Canto and Gurney, 1995; Wong et al., 1995). Loss of smell has been observed in ALS patients and excessive lipofuscin deposition has been noted in the mitral and tufted cells of their olfactory bulb (Elian, 1991; Hawkes et al., 1998). We explored here distribution of Ac-K123 SOD1 within the olfactory system.

**Figure 6:**
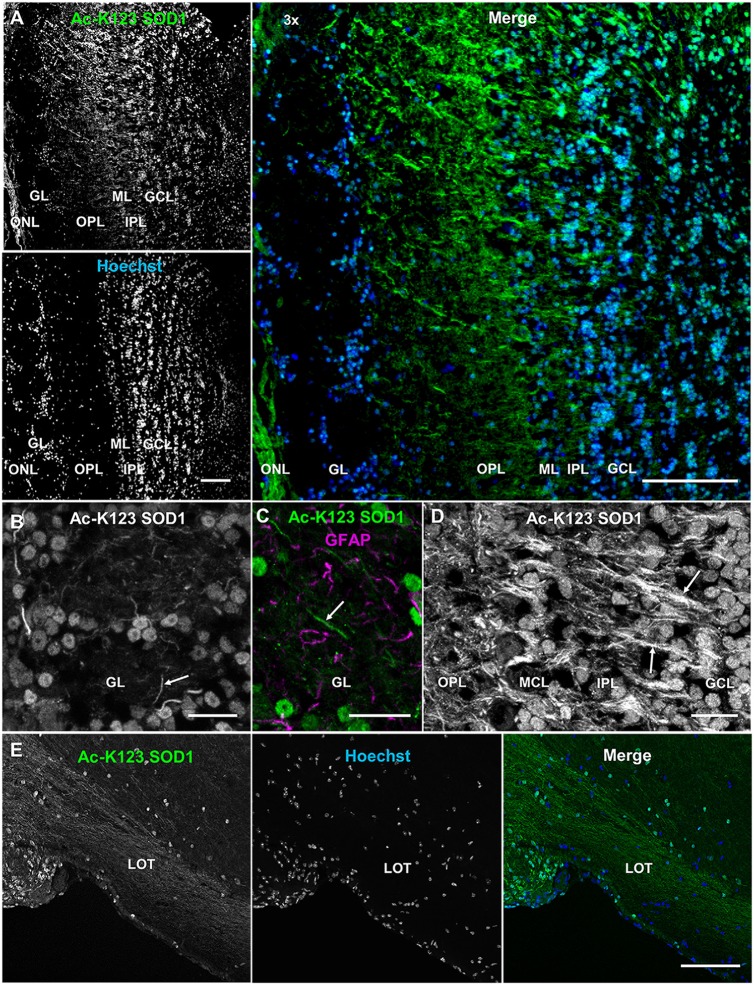
**Ac-K123 SOD1 within the olfactory bulb and lateral olfactory tract (LOT). (A)** Confocal micrograph (scale bar, 100 μm) showing Ac-K123 SOD1 (R26) immunostaining, Hoechst nuclear counterstaining and 3× merged image of the posterior main olfactory bulb in the coronal plane. Identified regions include olfactory nerve layer (ONL), glomerular layer (GL), outer plexiform layer (OPL), mitral cell layer (MCL), inner plexiform layer (IPL) and granule cell layer (GCL). ** (B)** Confocal micrograph (scale bar, 20 μm) of GL immunostained for Ac-K123 SOD1 (R26). An example of a neurite extending into the glomerulus is also labeled (arrow).** (C)** Confocal micrograph (scale bar, 20 μm) of an olfactory glomerulus core within the GL labeled for Ac-K123 SOD1 (R26) and glial fibrillary acidic protein (GFAP). An example of a neurite labeled with Ac-K123 SOD1 and not GFAP is shown (arrow). **(D)** Confocal micrograph of the OPL, ML, IPL and GCL immunostained for Ac-K123 SOD1 (R26; scale bar, 20 μm). Granule cell processes and tufted or mitral cell primary axons are labeled (arrows). **(E)** Sagittal view confocal micrograph (scale bar, 100 μm) of posterior olfactory bulb showing axonal tracts extending through the LOT in the area of the olfactory peduncle**.** Immunostaining of olfactory bulb was performed with antibodies against Ac-K123 SOD1 (R26) and nuclei were counterstained with Hoechst 33342.

Olfactory bulb coronal sections exhibited strong Ac-K123 SOD1 antibody staining of the olfactory nerve layer, the surface of the olfactory bulb formed by afferent projections of the olfactory sensory neurons (Figure 6A). Previous immunohistochemical analysis identified high SOD1 immunoreactivity in olfactory sensory neurons (Yon et al., 2008), which are responsible for odor detection through their odorant receptors. Axons from olfactory sensory neurons project toward the main olfactory bulb, where they synapse with second-order neurons, the mitral and tufted cells, in specialized compartments called glomeruli. The glomerular layer was void of staining with the exception of weak labeling in the cell bodies surrounding glomeruli (Figure 6A). Beyond the glomerular layer, Ac-K123 SOD1 staining was observed in processes spanning the outer plexiform layer (OPL), internal plexiform layer and granule cell layer (Figure 6A). Cell bodies of the granule cell layer also exhibited Ac-K123 SOD1 labeling (Figure 6A).

To better assess Ac-K123 SOD1 staining of the glomerular layer, we examined this region at higher magnification and identified Ac-K123 SOD1 distribution within the cell bodies of periglomerular cells, the small interneurons adjacent to the glomeruli, and processes within the olfactory glomeruli core (Figure 6B). These processes are probably neuronal, as they did not co-label with antibodies against GFAP, an astrocyte marker (Figure 6C). These Ac-K123 SOD1 positive neurites within the glomeruli core could be either axons of olfactory sensory neurons or dendrites of mitral and tufted cells.

A higher magnification view also showed Ac-K123 SOD1 staining of processes in the OPL (Figure 6D). This layer contains the somata of tufted cells, basal dendrites of tufted and mitral cells, and apical dendrites of granule cells. Within the mitral cell layer (MCL), crossing processes showed strong Ac-K123 SOD1 labeling, while large mitral cell bodies were weakly stained (Figure 6D). In the internal plexiform layer, through which axons of mitral and tufted cells course, staining was observed within processes (Figure 6D). Similar to the periglomerular interneurons, cells within the granule cell layer exhibited Ac-K123 SOD1 distribution within their somata (Figure 6D). These GABAergic granule cells, the principal interneurons of the olfactory bulb, modulate mitral and tufted cell activity by inhibitory feedback. In addition to the granule cell somata, Ac-K123 SOD1 was observed within processes of the granule cell layer (Figure 6D). Mitral and tufted cells project axons through this layer, eventually emerging from the caudal portion of the olfactory bulb to form the lateral olfactory tract (LOT). These efferent fibers of the LOT connect the olfactory bulb to several central brain regions via collateral branching (Schwob and Price, 1984).

A sagittal view of the posterior olfactory bulb identified Ac-K123 SOD1 within axonal tracts extending through the LOT in the area of the olfactory peduncle, the region connecting the olfactory bulb with the basal forebrain. From this view, Ac-K123 SOD1 was also observed in cell bodies of the olfactory bulb granule cell layer (Figure 6E).

In summary, olfactory bulb immunostaining demonstrated Ac-K123 SOD1 localization to cell bodies of interneurons and along processes of the major afferent neurons (olfactory sensory neurons) and efferent neurons (mitral and tufted cells).

### Ac-K123 SOD1 Is Present in Retinal Ganglion Cells

SOD1 plays a protective role in the retina, an area particularly susceptible to oxidative stress (Dong et al., 2006). SOD1 deficiency promotes injury of retinal neurons, and *Sod1*^−/−^ mice develop features of retinal degenerative disorders such as age-related macular degeneration and normal tension glaucoma (Imamura et al., 2006; Hashizume et al., 2008; Yuki et al., 2011). We performed immunohistochemical analysis of frozen mouse retinal tissue sections to compare Ac-K123 SOD1 levels within specific neuronal cells of the retina.

**Figure 7:**
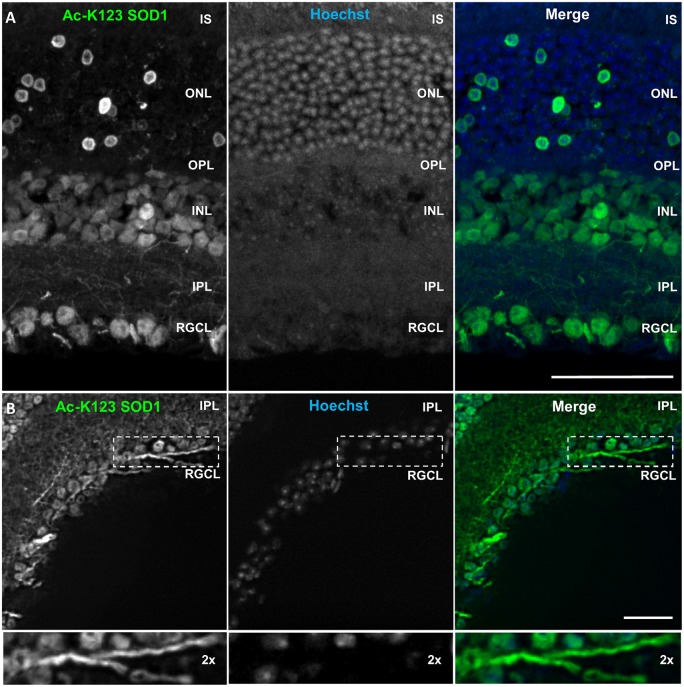
**Ac-K123 SOD1 localization to retinal ganglion cells (RGCs), outer and inner nuclear layer (INL) cell bodies and IPL neurites. (A)** Confocal micrograph (scale bar, 50 μm) of a vertical section through the mouse retina showing Ac-K123 SOD1 (R26) immunostaining with nuclear counterstaining by Hoechst 33342. Identified retinal layers include the inner segments of photoreceptor (IS), outer nuclear layer (ONL), OPL, INL, IPL and retinal ganglion cell layer (RGCL). **(B)** Fluorescence micrograph (scale bar, 50 μm) of a vertical section through the mouse retina demonstrating Ac-K123 SOD1 (R26) distribution within RGCL and IPL. Inset shows a 2× zoom of the boxed region containing a labeled RGC axon.

Immunostaining results showed strong Ac-K123 SOD1 detection in the retinal ganglion cell layer (RGCL; Figure 7A). This layer contains cell bodies of retinal ganglion cells (RGCs) and displaced amacrine cells. Because Ac-K123 SOD1 exhibited strong localization to both cell bodies and processes (Figure 7A), we sought out sections with intact processes to better assess Ac-K123 SOD1 labeling within the RGCL. Results from selected sections demonstrated Ac-K123 SOD1 distribution to somata and neurites of RGCs (Figure 7B). Closer examination of this layer indicated Ac-K123 SOD1 labeling of RGC axons (Figure 7B, inset).

Labeling was also observed within somata of the inner nuclear layer (INL; Figure 7A), which contains the neuronal cell bodies of bipolar, horizontal and amacrine cells. The somata of Müller cells, radial glia that span the entire retina, are also found in the INL. Ac-K123 SOD1 staining was evident within the neuronal processes of the inner plexiform layer (IPL; Figures 7A,B), the region between the RGCL and INL that contains the area of synaptic contact between the bipolar cell axons and the dendrites of the ganglion and amacrine cells.

The staining of the outer nuclear layer (ONL), which contains the cell bodies of rods and cones, was strong but less uniform than that of the RGCL and INL (Figure 7A). Because the density of rods is much greater than cones throughout most of the retina, we surmise that the relatively sparse number of Ac-K123 SOD1 positive ONL cell bodies are those of cone cells. This finding is of special interest because SOD1 is an important component of the antioxidant defense system of cones (Usui et al., 2011). Weaker Ac-K123 SOD1 labeling was observed in the OPL (Figure 7A), the small region between the INL and ONL where projections of rods and cones terminate and synapse with dendrites of bipolar cells.

In summary, the immunostaining results demonstrated that Ac-K123 SOD1 was localized to cell bodies within the INL and RGCL, as well as putative cone cells of the ONL. Ac-K123 SOD1 was also distributed along RGC axons and processes coursing through the IPL. Considering that previous histochemical analysis has shown diffuse cytoplasmic expression of SOD1 in the retina of wild-type mice (Imamura et al., 2006; Yuki et al., 2011), our findings suggest that SOD1 K123 acetylation may be important to specific cells or regions of the retina. It was previously shown that SOD1 is indispensable to RGCs and SOD1 deficiency causes RGC vulnerability and death (Yuki et al., 2011). SOD1 has also been shown to play an important neuroprotective role against excitotoxicity in RGCL and INL cells (Yuki et al., 2013). Because our immunostaining results indicated high levels of Ac-K123 SOD1 within these regions, it would be interesting to investigate if there is a correlation between SOD1 K123 acetylation and retinal neuron survival.

## Conclusion

During our investigation of SOD1 K123 acetylation, we generated rabbit polyclonal antibodies against this PTM and examined distribution of Ac-K123 SOD1 in the adult nervous system. In the cerebellum, strongest Ac-K123 SOD1 immunoreactivity was observed in glutamatergic granule cells. In the hippocampus, the somata of glutamatergic dentate granule cells and CA1 pyramidal cells were strongly stained for Ac-K123 SOD1. The strongest Ac-K123 SOD1 labeling of the olfactory bulb was along olfactory sensory neuron fibers and axons of glutamatergic tufted and mitral cells. In the retina, the glutamatergic ganglion cells exhibited the highest levels of Ac-K123 SOD1. INL cell bodies and glutamatergic ONL cone cells also exhibited considerable Ac-K123 SOD1 immunoreactivity. These results suggest that SOD1 K123 acetylation is largely restricted to specific functionally distinct neuronal subtypes.

Another key finding was the localization of Ac-K123 SOD1 within processes of major afferent and efferent neurons. In the hippocampal formation, axonal tracts of the subiculum and fimbria were strongly labeled, as were apical dendrites of the efferent CA1 pyramidal cells. Strong Ac-K123 SOD1 labeling was also noted within mitral and tufted cell axonal projections of the olfactory bulb, along fibers coursing through the LOT, and in the efferent axons of RGCs. The propensity of Ac-K123 SOD1 to localize within long axonal projections of afferent and efferent neurons, as opposed to the shorter processes of interneurons, merits further study. It is possible that Ac-K123 SOD1 interacts with proteins specific to these axons, such as phosphorylated neurofilament heavy chain proteins, which are predominantly found in larger axons.

Finally, identification of Ac-K123 SOD1 in cells associated with CSF is another interesting finding. Ac-K123 SOD1 was highly localized to CP cuboidal cells and ependymal cells of the ventricles and central canal, the cells responsible for CSF production and circulation within the brain and spinal cord.

Our observations lay the foundation for future investigations into the role of acetylation in physiology and pathophysiology of the nervous system.

## Author Contributions

MK performed cell culture experiments and western blots (Figures 1A, 2A–E), tissue embedding (Figures 1B, 5C), cryosectioning (Figures 1B, 2H,I, 3D, 4C,D, 5C, 6A–D, Supplementary Figure 1), immunostaining (Figures 1B, 2F,H,I, 3A,C,D, 4A–D, 5A–C, 6A–F, Supplementary Figure 1), confocal imaging (Figures 2F,H,I, 3B–D, 4B–D, 5A–C, 6A–E), site-directed mutagenesis (Supplementary Figure 1), data analysis, figure preparation and writing of manuscript. AKK performed cryosectioning (Figures 6A–D), immunostaining (Figure 7A), confocal imaging (Figures 1B, 7A), wide-field fluorescence microscopy (Figures 3A, 4A, 7B), image montage stitching (Figures 3A, 4A) and contributed to preparation of figures, legends and methods. SLB performed tissue embedding and sectioning (Figures 2H,I, 3A–D, 4A,B, 5A,B, 6E, 7A,B, Supplementary Figure 1) and immunostaining (Figure 7B). RSS performed SOD1 protein purification (Supplementary Figure 2) and immunostaining (Figure 3B). ABK participated in manuscript development, writing and editing. HAH performed immunostaining (Figure 2F). BB performed site-directed mutagenesis (Figure 2A) and SOD activity assays (Supplementary Figure 2). WS performed site-directed mutagenesis (Figure 2A). T-TH provided the *Sod1^+/+^* and *Sod1^−/−^* frozen mouse brains. EB-W conceived and supervised all aspects of the project. All authors read and approved the final manuscript.

## Conflict of Interest Statement

The authors declare that the research was conducted in the absence of any commercial or financial relationships that could be construed as a potential conflict of interest.

## Footnotes

^1^http://alsod.iop.kcl.ac.uk/

## Abbreviations

Ac-K123 SOD1: lysine 123-acetylated SOD1
Ac-peptide: acetylated K123 peptide
AD: Alzheimer’s disease
ALS: amyotrophic lateral sclerosis
BC: basket cells
CA1: cornus ammonis field 1
CA3: cornus ammonis field 3
CC: corpus callosum
CP: choroid plexus
CSF: cerebrospinal fluid
ECL: enhanced chemiluminescence
ELISA: enzyme-linked immunosorbent assay
FBS: fetal bovine serum
GCL: granular cell layer
GFAP: glial fibrillary acidic protein
HDAC: histone deacetylase
INL: inner nuclear layer
IPL: inner plexiform layer
IPTG: isopropyl β-D-1-thiogalactopyranoside
K123: lysine 123
LB: Luria Bertani broth
LOT: lateral olfactory tract
MCL: mitral cell layer
ML: molecular layer
NA: numeric aperture
NAM: nicotinamide
OCT: optimal cutting temperature
ONL: outer nuclear layer
OPL: outer plexiform layer
PBS: phosphate buffered saline
PCL: Purkinje cell layer
PTM: post-translational modification
RGCL: retinal ganglion cell layer
SC: stellate cells
SDS: sodium dodecyl sulfate
SLM: stratum lacunosum-moleculare
SOB: Super Optimal Broth
SOD1: superoxide dismutase 1
SOD1 K123Q: acetyl-mimetic mutant of SOD1
SOD1 K123R: deacetyl-mimetic mutant of SOD1
SP: stratum pyramidale
SR: stratum radiatum
TSA: trichostatin A.
