# Supplementary material for: SOD1 Lysine 123 Acetylation in the Adult Central Nervous System
Source: Front Cell Neurosci. 2016 Dec 20;10:287. doi: 10.3389/fncel.2016.00287 (PMC5167747; doi:10.3389/fncel.2016.00287)
Supplement: Supplementary file 1 [file DataSheet_1.pdf]

Supplementary Material

SOD1 Lysine 123 Acetylation in the Adult Central Nervous System

Running Title: SOD1 Lysine 123 Acetylation

Michael Kaliszewski[1], Austin Kennedy[1], Shelby Blaes[1], Robert Shaffer[1], Andrew B. Knott[1], Wenjun Song[1,2], Henry Hauser[1], Blaise Bossy[1], Ting-Ting Huang[3,4], Ella Bossy-Wetzel[1,]*

[1] Burnett School of Biomedical Sciences, College of Medicine, University of Central Florida, Orlando, FL, USA
[2] Yale School of Forestry and Environmental Studies, Yale University, New Haven, CT, USA
[3] Department of Neurology and Neurological Sciences, Stanford University School of Medicine, Stanford, CA, USA
[4] Geriatric Research, Education, and Clinical Center, VA Palo Alto Health Care System, Palo Alto, CA USA

Correspondence: Ella Bossy-Wetzel (ella.bossy-wetzel@ucf.edu)

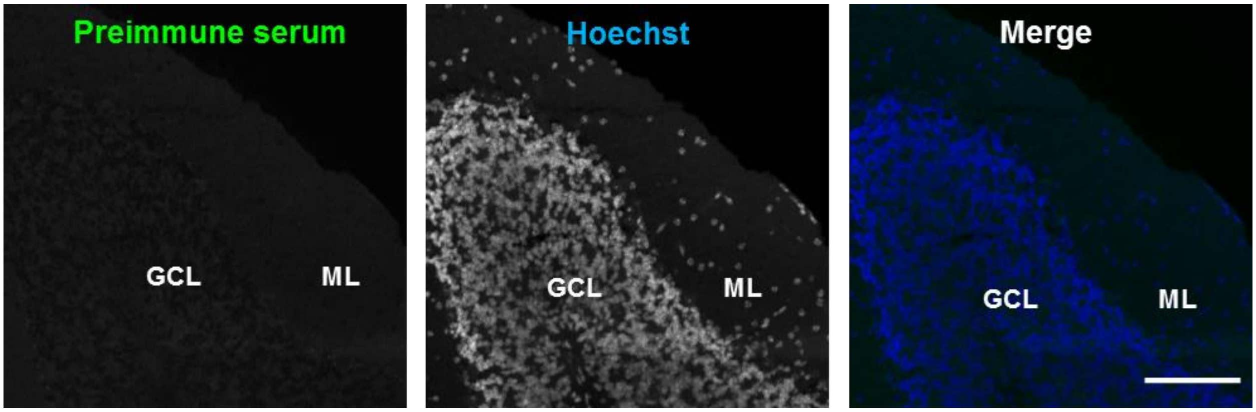

Supplementary Figure 1. Incubation with rabbit preimmune serum. Confocal micrograph (scale bar, 100 µm) of a mouse cerebellum sagittal section incubated with preimmune serum collected from rabbit 26 prior to immunization against Ac-K123 SOD1. Nuclei were counterstained with Hoechst 33342. The granular cell layer (GCL) and molecular layer (ML) of the cerebellar cortex are identified.

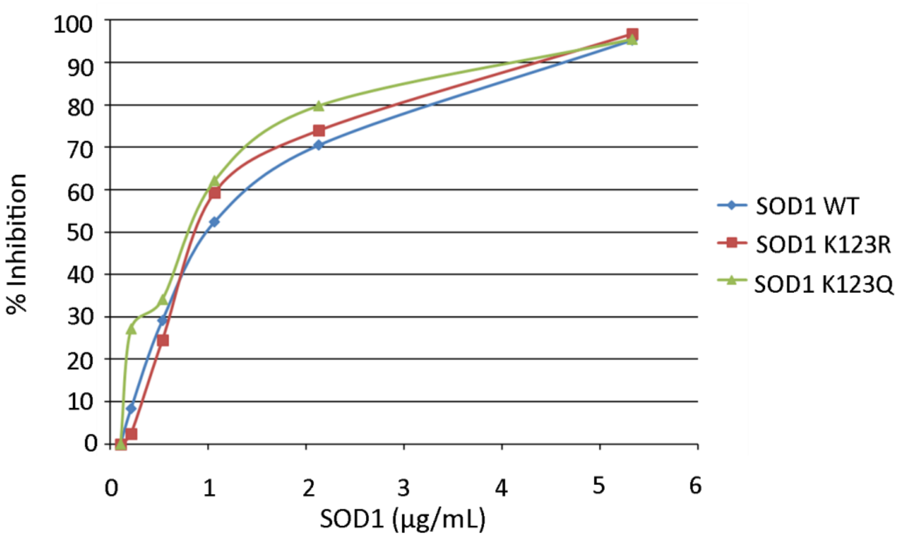

Supplementary Figure 2. SOD activity assay. Superoxide dismutase activity of SOD1 WT, SOD1 K123R, and acetyl-mimetic SOD1 K123Q measured showing inhibition rate (%) of WST-1 reduction by superoxide anion per concentration of purified recombinant SOD1 protein (microgram per mL).
